# Evaluation of the therapeutic potential of novel nanoparticle formulations of glutathione and virgin coconut oil in an experimental model of carbon tetrachloride-induced liver failure

**DOI:** 10.1186/s40360-024-00795-x

**Published:** 2024-10-08

**Authors:** Essmat A. H. Allam, Madeha H. A. Darwish, Nasser S. Abou Khalil, Shimaa H. A. Abd El-Baset, Mohamed Abd El-Aal, Ahmed Elrawy, Ahmed A. N. Ahmed, Mahmoud S. Sabra

**Affiliations:** 1 Department of Pharmacology and Toxicology, Faculty of Pharmacy, Assiut University, Assiut, 71526 Egypt; 2 Department of Animal and Poultry Behavior and Management, Faculty of Veterinary Medicine, Assiut University, Assiut, 71516 Egypt; 3 Department of Animal Physiology and Biochemistry, Faculty of Veterinary Medicine, Badr University, Assiut, Egypt; 4 Department of Medical Physiology, Faculty of Medicine, Assiut University, Assiut, 71516 Egypt; 5 Department of pathology and clinical pathology, Faculty of Veterinary Medicine, Sphinx University, Assiut, Egypt; 6 Chemistry Department, Faculty of Science, Assiut University, Assiut, 71516 Egypt; 7 Pharmacology Department, Faculty of Medicine, Al-Azhar University, Assiut Branch, Assiut, 71526 Egypt; 8 Department of Pharmacology, Faculty of Veterinary Medicine, Assiut University, Assiut, 71526 Egypt; 9 Pharmacology Department, Faculty of Veterinary Medicine, Badr University, Assiut, Egypt

**Keywords:** Carbon tetrachloride, ALF, Nanoparticles, VCO, IL-1β, NF-κB

## Abstract

**Background:**

Acute liver failure (ALF) is a critical condition characterized by rapid liver dysfunction, leading to high mortality rates. Current treatments are limited, primarily supportive, and often require liver transplantation. This study investigates the potential of a novel nanoparticle formulation of glutathione (GSH) and virgin coconut oil (VCO) alone and in combination to enhance therapeutic outcomes in a rat model of ALF induced by orogastric carbon tetrachloride (CCl_4_).

**Methods:**

The study employed adult male Albino rats divided into ten groups, with ALF induced via a single oral dose of CCl_4_. Various treatment regimens were administered over seven days, including conventional and nanoparticle forms of GSH and VCO and their combinations. The efficacy of treatments was evaluated through biochemical analysis of liver function markers, oxidative stress indicators, inflammatory biomarkers, and histopathological examinations. Nanoparticles were synthesized using established methods, and characterization techniques were employed to ensure their quality and properties.

**Results:**

The nanoparticle formulations significantly improved liver function, as indicated by reduced serum levels of alanine aminotransferase and aspartate aminotransferase, alongside decreased oxidative stress markers such as malondialdehyde. Furthermore, they reduced tumor necrosis factor alpha and interleukin-1 beta inflammatory markers. Histological analysis revealed reduced hepatocellular necrosis and inflammation in treated groups compared to controls. Also, decreased nuclear factor-kappa B was detected by immunohistochemical analysis.

**Conclusion:**

The findings show that the nanoparticle mixture of GSH and VCO effectively reduces liver damage in ALF. This suggests a promising drug-based approach for improving liver regeneration and protection. This innovative strategy may pave the way for new therapeutic interventions in the management of ALF.

**Supplementary Information:**

The online version contains supplementary material available at 10.1186/s40360-024-00795-x.

## Introduction

Acute liver failure (ALF) is an uncommon, yet potentially lethal medical condition marked by the sudden emergence of significant liver impairment in patients who do not have any pre-existing liver ailments. It is characterized by the occurrence of coagulopathy (with an internationally normalized ratio [INR] of ≥ 1.5) and hepatic encephalopathy within 26 weeks of the initial symptoms [1]. In developed countries, ALF has an estimated annual incidence of 1–6 cases per 100,000 people, with a high mortality rate ranging from 30 to 90% without liver transplantation [2].

The pathophysiology of ALF involves massive hepatocellular necrosis, leading to the impairment of liver functions, including protein synthesis, metabolism, and detoxification. This outcomes in the deposit of toxic substances, such as ammonia, and the development of complications like cerebral edema and multi-organ failure [3]. Current treatments for ALF are primarily supportive, aimed at managing complications and providing liver transplantation when feasible [4]. However, the availability of donor organs is limited, and the use of immunosuppressive drugs after transplantation can lead to adverse effects [5]. Moreover, there is a lack of effective pharmacological interventions that can directly target the underlying mechanisms of liver injury and promote regeneration.

Glutathione (GSH) is a tripeptide with potent antioxidant and detoxifying properties, plays a vital function in preserving the balance of cellular redox levels [6]. The pathogenesis of various liver diseases, including ALF, has implicated GSH depletion. Studies have established the antioxidant, anti-inflammatory, and hepatoprotective effects of virgin coconut oil (VCO), a natural product rich in medium-chain fatty acids [7]. The mechanisms underlying these beneficial effects include the modulation of oxidative stress, inflammation, and apoptosis pathways [8].

Nanoparticle (NP) forms of GSH and VCO are better than their regular forms in a number of ways, including better bioavailability, targeted delivery, and long-lasting release [9, 10]. NP can enhance the solubility and stability of these compounds, facilitating their absorption and distribution to target tissues [11, 12]. Additionally, NP can be engineered to selectively accumulate in the liver, potentially increasing therapeutic efficacy while minimizing systemic side effects [13]. Regarding the chitosan (CS) and ferric oxide (Fe_3_O_4_) utilized in our research. A recent review focuses on nanoparticles composed of chitosan and its derivatives, intended for liver targeting [14]. Additionally, a review demonstrated liver imaging via superparamagnetic iron oxide nanoparticles [15].

Carbon tetrachloride (CCl_4_) is a well-established hepatotoxin widely used to induce experimental models of ALF in rodents [16]. CCl_4_ administration causes reactive metabolites and oxidative stress, resulting in hepatocellular necrosis and liver failure. The new oral CCl_4_-induced moderate liver damage model closely resembles the pathophysiological features seen in human ALF, which makes it a good model for testing possible therapeutic interventions [17, 18].

Our investigation, which used male rats, finding revealed that eight of ten fields (reproduction and immunology being the exceptions) have male bias: general biology, neuroscience, physiology, pharmacology, endocrinology, behavioral physiology, behavior, and zoology. However, important discoveries in fundamental and therapeutically relevant research should result from the inclusion of both sexes in animal research investigations. It is necessary to test female rats throughout the estrus cycle since they are more variable than male rats and include both sexes increases variability [19].

The goal of this study is to look into how conventional and NP forms of GSH and VCO protect against ALF caused by CCl_4_ in rats. The antioxidant and liver-protecting properties of GSH and VCO, along with the possible benefits of NP delivery systems, can lead to better therapeutic outcomes and new ideas for dealing with ALF.

## Materials and methods

### Animals and the development of ALF

The investigation was conducted in accordance with the ethical treatment criteria for laboratory animals, as specified in the Guide for the Care and Use of Laboratory Animals and the ARRIVE recommendations. The Institutional Animal Ethics Committee granted prior consent (approval no. 06/2024/0225). Male albino rats, aged 10–12 weeks, were acquired from the Animal Facility at the Faculty of Veterinary Medicine, Assiut University, Egypt. The age was selected because the rodent was an adult. But in numerous studies, the term “adult” was used to refer to a range of ages between six and twenty weeks [20]. The rats had an average weight of 250 g and were kept in a room with good air circulation. The room’s conditions were carefully managed, with temperatures ranging from 27 to 32 °C and a 12-hour cycle of light and darkness (lights on from 5:00 a.m. to 7:00 p.m.). The rodents were given unrestricted access to regular rodent chow (containing 21% protein, 3% fat, 5% fiber, 8% ash, 0.8% calcium, 0.4% phosphorus, and 1.3% silica w/w) and drinking water.

To cause moderate ALF, the rats were given a single oral dose of CCl_4_ at a concentration of 2.5 mL/kg body weight. The solution was made up of 50% CCl_4_ and olive oil [18, 21].

### Animals’ groups

A total of ten groups were included in the study, each comprising six rats. VCO was administered orally (p.o.) at a dose of 15 mL/kg body weight, following previously established protocols [22]. GSH was given intraperitoneally (i.p.) at a dose of 100 mg/kg body weight. It has been demonstrated that giving rats glutathione injections twice a week for 13 weeks at a level of 248 mg/kg may have potentially hazardous effects [23]. In light of this the dose of GSH at a dose of 100 mg/kg body weight was chosen according to our previous work [22]. Furthermore, a prior study found that rats were given a single oral dosage of 5000 mg/kg of VCO in the acute toxicity experiment; for the sub-chronic and chronic investigations, the doses were 175, 550, and 2000 mg/kg. These findings suggest that long-term usage of VCO is safe [24]. In light of this the dose of VCO at a dose of 15 ml/kg body weight was chosen according to our previous work [22].

After the creation of the acute hepatic failure model, medications were provided to all groups for a duration of 7 days. Drug-loaded nanoparticles were administered at half the dosage of conventional ones.

Group I (Control) functioned as the control group, where they were given 1 mL/kg body weight of olive oil through p.o. and normal saline through p.o. for a duration of 7 days.

Group II (CCL_4_) was induced with ALF by a single oral dose of CCl_4_ at 2.5 mL/kg body weight (50% in olive oil), 24 h before sacrifice.

Group III (FO) received CCl_4_-induced ALF, using Fe_3_O_4_ NPs (7.5 ml/kg, p.o.) as carriers for 7 days.

Group IV (CS) received CCl_4_-induced ALF, using CS-based NPs (50 mg/kg, i.p.) as carriers for 7 days.

Group V (VCO) was given CCl_4_ and VCO (15 mL/kg p.o.) for 7 days.

Group VI (GSH) received CCl_4_ and GSH (100 mg/kg i.p.) for 7 days.

Group VII (O) was administered a combination of CCl_4_, GSH (100 mg/kg i.p.), and VCO (15 mL/kg p.o.) for 7 days.

Group VIII (Nano-VCO) received CCl_4_ and VCO-loaded Fe_3_O_4_ NPs (7.5 mL/kg p.o.) for 7 days.

Group IX (Nano-CS) was given CCl_4_ and GSH-loaded CS NPs (50 mg/kg i.p.) for 7 days.

Group X (Nano-O) received CCl_4_, GSH-loaded CS NPs (50 mg/kg i.p.), and VCO-loaded Fe_3_O_4_ NPs (7.5 mL/kg p.o.) for 7 days.

### Materials

The substances examined in this investigation, together with their molecular formulae and sources, are presented in Table 1.

**Table 1:** The materials utilized, along with their molecular formulas and the suppliers

Substance	Formula	Source
Anhydrous ferric chloride	FeCl_3_	Alpha chemicals (Cairo, Egypt)
Ammonium ferrous sulphate heptahydrate	(NH_4_)_2_Fe(SO_4_)_2_·6H_2_O)	Alpha chemicals (Cairo, Egypt)
Sodium hydroxide	NaOH	Alpha chemicals (Cairo, Egypt)
Virgin coconut oil (VCO)	-	Local market in Assiut, Egypt, and authenticated by a medicinal plant expert at the Processing and Extraction Unit of Medicinal Plants, Faculty of Agriculture, Assiut University, Egypt.
Chitosan (CS)	Low molecular weight	Sigma-Aldrich, USA
Sodium tripolyphosphate (TPP)	Na_5_P_3_O_10_	Qualikems, India
N-acetylcysteine (NAC)	C_5_H_9_NO_3_S	AKSCI, USA
Acetic acid (AA)	C_2_H_4_O_2_	Alpha Chemicals, Cairo, Egypt

### Synthesis of Fe3O4 nanoparticles

Fe3O4 nanoparticles were produced using the co-precipitation technique [1–3]. First, a solution (A) was made by dissolving anhydrous FeCl3 (21 g) and (NH4)2Fe(SO4)2·6H2O (25.4 g) in 500 mL of bi-distilled water. Solution (B) was produced by dissolving 50 g of NaOH in 500 mL of water. Solution (A) was then gradually added dropwise to solution (B) with vigorous stirring. The resulting mixture was heated at 60 °C for 2 h with constant stirring and then allowed to cool to room temperature. The black precipitate that developed was separated using a magnet, rinsed with bi-distilled water and ethanol, and dried overnight at 60 °C.

### Synthesis of Fe3O4@VCO nanocomposite

The Fe_3_O_4_@VCO nanocomposite was prepared by adding 27.6 g of the Fe_3_O_4_ nanoparticles to 30 mL of pure coconut oil and stirring for 30 min. An ultrasonic generator was used to disperse the Fe_3_O_4_ nanoparticles in the oil and reduce agglomeration. The sonication was carried out for 1 h. The oil samples were then permitted to rest for 30 min at room temperature before being dried in an oven at 85 °C for 48 h.

### Synthesis of CS NPs

Chitosan NPs were produced using the ionotropic gelation process, following the procedure reported by Ayodele, Olanipekun [25] with some changes. Initially, 2.6 g of pure CS was carefully weighed and dissolved in 160 ml of 2% acetic acid (AA) solution. Separately, 6 g of sodium tripolyphosphate was weighed and dissolved in 160 ml of distilled water. The sodium tripolyphosphate solution was then added dropwise to the CS solution, and the resulting mixture was let to stand for 24 h to ensure complete equilibration. Subsequently, the mixture was filtered, and the residue was washed multiple times with distilled water. Finally, the residue was dried in an oven at 45 °C, resulting in the creation of CS NPs.

### Synthesis of GSH@CS nanocomposite

To prepare the GSH@CS nanocomposite, 0.1 g GSH was dissolved in a small amount of distilled water. The GSH solution was then carefully mixed with 0.4 g of the previously synthesized CS NPs until a homogeneous paste was formed [26, 27]. The mixture was then dried in an oven at 50 °C for 24 h.

### Characterization techniques

The nanocomposites that were produced were subjected to examination utilizing a variety of procedures, as outlined in Table 2.

**Table 2:** Outlines the methods of characterization employed in this investigation

Technique name	Instrument
X-ray diffraction (XRD)	Philips diffractometer, model PW 2103/00
Fourier transform infrared (FTIR)	Nicolet spectrophotometer, model 6700
Transmission electron microscopy (TEM)	JEOL Model JSM-5400 LV

### Collection of blood and tissue samples

After an overnight fast of approximately 12–14 h, blood samples were collected from the retro-orbital venous plexus of anesthetized rats via the eye canthus. Prior to sacrifice, rats were euthanized following an approved protocol: rats were deeply anesthetized by inhalation of 5% isoflurane, and once unresponsive to head and limb stimulation, they were euthanized by cervical dislocation. Death was confirmed by the absence of breathing and lack of response to systemic stimulation for at least 10 s post-dislocation [28].

Blood samples were collected in plain tubes and centrifuged at 4000 rpm for 15 min to obtain serum, which was stored at -20 °C until further analysis. Immediately after euthanasia, the lungs were excised, rinsed with 0.9% saline solution, and processed for histological examination.

Liver tissues were minced and homogenized (10% w/v) in ice-cold potassium phosphate buffer (0.1 M, pH 7.4). The homogenate was centrifuged at 3000×*g* for 10 min at 4 °C, and the resulting supernatant was used for the assessment of oxidative stress markers, antioxidant levels, and inflammatory mediators. Additional liver tissue samples were preserved for histopathological and immunohistochemical analyses.

### Determination of liver function markers

#### Detection of serum aspartate aminotransferase (AST) activity

The AST activity, a marker of liver function, was determined using a commercial AST enzyme activity assay kit. The test is based on the idea that AST, an enzyme that needs pyridoxal phosphate (PLP), moves an amino group from aspartate to α-ketoglutarate, which makes oxaloacetate and glutamate. After being broken down, the oxaloacetate turns into a colorimetric product whose intensity depends on how much AST enzyme activity is in the sample. The kinetic technique adopted was initially outlined by Reitman and Frankel [29].

#### Assessment of serum alanine aminotransferase (ALT) activity

The activity of ALT, another liver function measure, was evaluated using a kinetic test established by Reitman and Frankel [29]. ALT, which is also called glutamate-pyruvate transaminase (GPT), is an enzyme that needs PLP to work. It moves an amino group from alanine to α-ketoglutarate and back again, making pyruvate and glutamate. The pyruvate formed in this reaction is then converted into a colorimetric product, with an intensity proportional to the sample’s ALT enzymatic activity.

#### Determination of serum albumin level

The serum albumin (ALB) concentration was determined using a colorimetric assay described by Doumas et al. [30]. The assay principle relies on the formation of a colored complex between ALB and bromcresol green at an acidic pH of 3.8. The intensity of the colorful complex, as determined photometrically, is proportional to the ALB content in the sample.

#### Determination of serum alkaline phosphatase (ALP) activity

Serum ALP activity was assessed by a colorimetric method published by Bowers and McComb [31]. This assay is based on ALP’s enzymatic hydrolysis of phenyl phosphate, which releases phenol. The amount of freed phenol is then measured using a colorimetric reaction with potassium ferricyanide and 4-aminophenazone. The rate of color development is directly related to the ALP activity present in the serum sample.

#### Serum gamma-glutamyl transferase (GGT) level determination

The kinetic technique described by Bergmeyer et al. [32] was utilized to measure serum GGT activity. In this test, GGT helps transport the gamma-glutamyl group from the donor substance L-gamma-glutamyl-3-carboxy-4-nitroanilide to the acceptor substance glycylglycine. This generates 3-carboxy-4-nitroaniline. The rate of rise in absorbance owing to the production of 3-carboxy-4-nitroaniline is directly related to the GGT activity in the serum sample.

#### Assessment of serum bilirubin levels

Serum bilirubin was detected using the colorimetric technique described by Rutkowski and E Baare [33]. The test was conducted in line with the reagent kits, which derive from the reaction between bilirubin and the diazonium salt of sulfanilic acid generating azobilirubin with a maximum absorption at 535 nm in an acidic environment. Whereas only conjugated bilirubin reacts in the absence of dimethylsulfoxide (DMSO), total bilirubin participates in the reaction in the presence of DMSO.

### Evaluation of liver tissue lipid peroxidation

The level of malondialdehyde (MDA), a marker of lipid peroxidation and oxidative stress, was determined in liver tissue homogenates using the thiobarbituric acid assay described by Ohkawa et al. [34]. The MDA concentration in liver homogenate was assessed via the Bradford technique [35]. This colorimetric assay involves the reaction of MDA with thiobarbituric acid to form a colored adduct, which is quantified spectrophotometrically [36].

### Evaluation of liver tissue antioxidant enzyme activities

#### Estimation of glutathione peroxidase (GPx) activity

Using Paglia and Valentine’s coupled enzyme assay approach, GPx’s activity in liver tissue homogenates was quantified [37]. This assay combines the tissue homogenate with a reaction mixture including GSH, GSH reductase, and NADPH. A substrate for the enzymatic process is hydrogen peroxide. Measuring the decrease in absorbance at 340 nm, the rate of NADPH oxidation is exactly proportional to the GPx activity of the sample.

### Determination of liver tissue inflammatory markers

#### Estimation of tumor necrosis factor alpha level

Tumor necrosis factor alpha (TNF-α) levels in rat liver tissues were quantitatively measured using commercial enzyme-linked immunosorbent assay (ELISA) kits, following the manufacturer’s procedure. The assay procedure began by adding rat liver tissue samples and TNF-α standards to monoclonal antibody-precoated 96-well plates. Subsequently, biotinylated detection antibodies specific to TNF-α were added. The signal was amplified using an Avidin-Biotin-Peroxidase Complex (ABC), and the reaction was visualized by adding a chromogenic substrate, resulting in a color change from blue to yellow upon the addition of a stop solution. The optical density of each well was measured using a UV/visible spectrophotometer at a specified wavelength to determine the concentration of TNF-α [38, 39].

#### Estimation of interleukin-1 beta level

Concentrations of interleukin 1 beta (IL-1β) were measured in rat liver tissues using ELISA kits designed for rat IL-1β. The assay involved coating the wells of a microplate with monoclonal antibodies against rat IL-1β. After incubating with liver tissue samples, bound IL-1β was detected with a biotinylated secondary antibody specific to IL-1β. Following the addition of an ABC reagent and a subsequent substrate solution, the resulting colorimetric change was quantified by measuring the absorbance at a specific wavelength. This absorbance is exactly related to the amount of IL-1β present in the samples [40].

### Histopathological evaluation

Liver tissue samples were taken and treated in 10% neutral buffered formalin to preserve cellular architecture. Following fixation, tissues were dehydrated in a graded sequence of alcohols, cleaned in xylene, and embedded in paraffin wax. Sections of 5 microns in thickness were cut and placed on slides. For histological investigation, slices were stained with hematoxylin and eosin (H&E), which emphasizes cellular and tissue architecture under a light microscope [41].

### Nuclear factor-kappa B immunohistochemistry in liver tissue

Immunohistochemical examination was done to detect nuclear factor-kappa B (NF-κB) in liver tissues from ten experimental groups. Tissue sections, four millimeters thick, were first deparaffinized and rehydrated. Endogenous peroxidase activity was inhibited using hydrogen peroxide in methanol. Antigen retrieval was aided by microwaving the slices in citrate buffer (pH 6.0). Sections were then treated with monoclonal anti-NF-κB antibodies (Thermo Fisher Scientific, USA) at room temperature. Detection was done utilizing the UltraVision Detection System (Thermo Scientific), which involves streptavidin peroxidase conjugation followed by diaminobenzidine (DAB) as the chromogen. The slides were counterstained with hematoxylin to increase nuclear contrast. Quantification of NF-κB immunopositivity was accomplished by counting the number of immunopositive cells in five random microscopic areas per slide. The average number of positive cells per field was computed, and the mean ± standard error (SE) for each group was determined [42].

### Statistical analysis

The statistical evaluation of the data began by validating the normal distribution of all variables using the Shapiro-Wilk normality test. Given the normal distribution, parametric approaches were utilized for subsequent studies. Differences among the experimental groups were analyzed using a one-way ANOVA, followed by Tukey’s multiple comparisons test to identify significant differences between groups. All statistical analyses were performed using GraphPad Prism software (version 8.0.2). A *p*-value < 0.05 was judged statistically significant.

## Results

### Characterization, TEM micrograph, XRD and FTIR of Fe3O4-VCO, GSH-CS nanocomposite

The XRD patterns of both pure Fe_3_O_4_ nanoparticles and the Fe_3_O_4_-VCO nanocomposite are shown in Supplementary (Fig. S1) and Supplementary (Fig. S2) on the other hand, presents the FTIR spectra of the pure Fe_3_O_4_ nanoparticles and the Fe_3_O_4_-VCO nanocomposite. The TEM micrograph and the particle size distribution of the Fe_3_O_4_-VCO nanocomposite are displayed in Supplementary (Fig. S3) according to our previous study [22].

The XRD patterns and the FTIR spectra of the CS nanoparticles and the GSH-CS nanocomposite are shown in Supplementary (Fig. S4a, b), respectively. Additionally, the TEM image and the particle size distribution of the GSH-CS nanocomposite is presented in Supplementary (Fig. S4c) according to our previous study [22].

### The impact of virgin coconut oil, glutathione, and their nanoparticle derivatives on the liver function markers

**Fig. 1:**
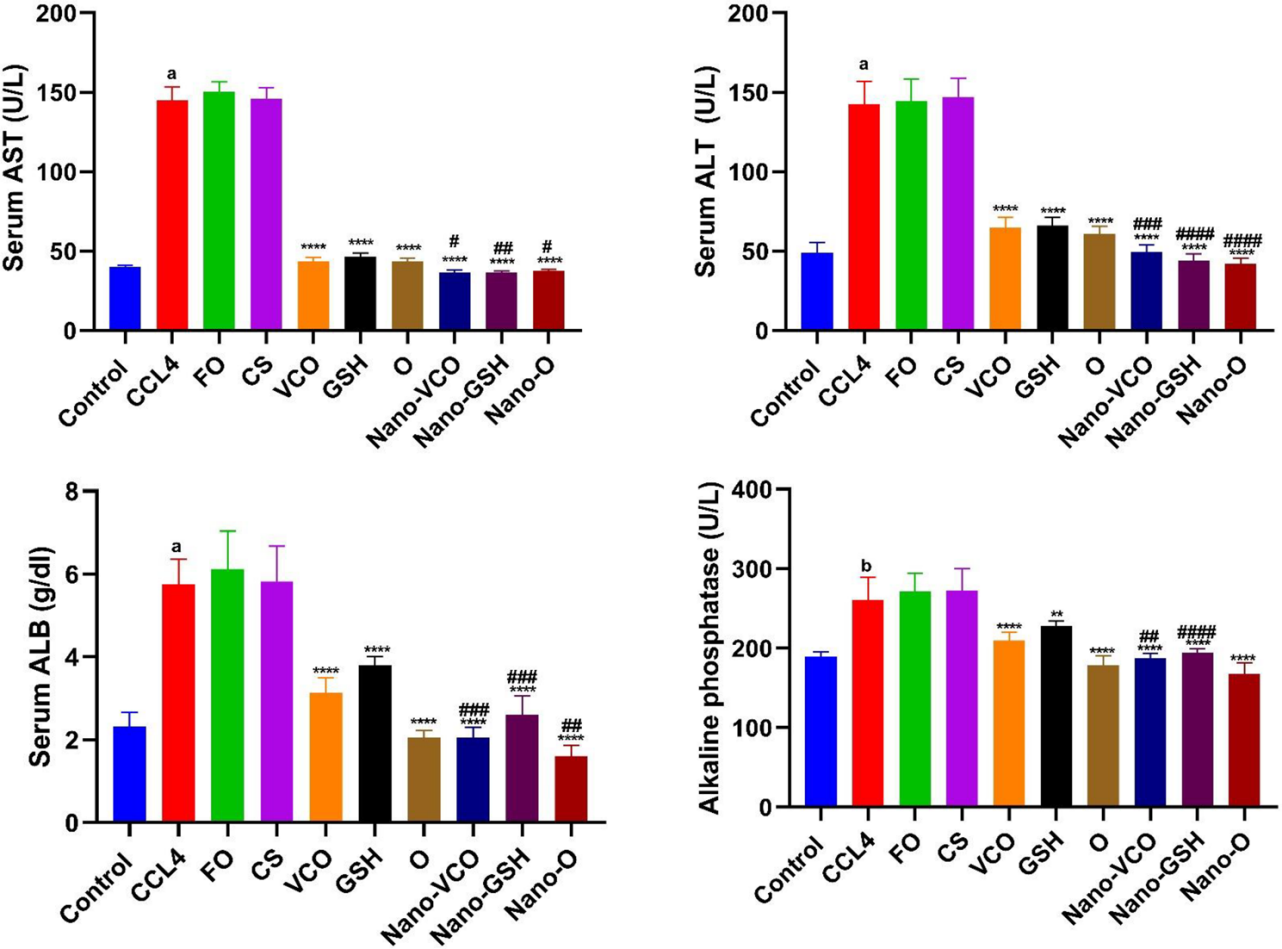
The impact of different forms of glutathione (GSH) and virgin coconut oil (VCO), including conventional, nanoparticle, and combination forms, on blood levels of aspartate aminotransferase (AST), alanine transaminase (ALT), albumin (ALB), and alkaline phosphatase in rats with acute liver failure induced by carbon tetrachloride (CCl_4_). Data are the means ± SEM (*n* = 6). ^b^*p* < 0.001 and ^a^*p* < 0.0001 as compared with the control group. **p < 0.01 and ****p < 0.0001 as compared to the CCl_4_-treated group. ^#^*p* < 0.05, ^##^*p* < 0.01, ^###^*p* < 0.001 and ^####^*p* < 0.0001 when contrasted with the related nanoparticle category. *Note FO* ferric oxide, *CS* chitosan, *Nano-VCO* virgin coconut oil nanoparticles, *Nano-GSH* glutathione nanoparticles, *O* virgin coconut oil-glutathione combinations, *Nano-O* virgin coconut oil-glutathione nanoparticle combinations

The group that was exposed to CCl_4_ had much higher levels of the liver enzymes AST (df = 10, F = 49.033), ALT (df = 10, F = 4.812), ALB (df = 10, F = 3.136), and ALP (df = 10, F = 24.23) than the negative control group (*p* < 0.0001). In rats treated with CS and Fe_3_O_4_ NPs, serum AST, ALT, ALB, and ALP levels were not significantly different from rats with liver failure (F_2,15_ = 0.1426, F_(2,15)_ = 0.1628, F_(2,15)_ = 0.3514, and F_(2,15)_ = 0.3849, respectively). Also, researchers looked at CCl_4_-liver failure induced rats and compared them to those given traditional or NPs with GSH, VCO, or a mix of these substances. The NPs-containing groups had much lower levels of AST (F_(6,35)_ = 123.9), ALT (F_(6,35)_ = 145.5), ALB (F_(6,35)_ = 91.75), and ALP (F_(6,35)_ = 31.60) (*p* < 0.0001). Compared to their conventional forms, the decrease was particularly noticeable in rats given NP versions of GSH, VCO, and their combination (Fig. 1).

### The impact of coconut oil, glutathione, and their nanoparticle formulations alone or in combination on blood bilirubin (direct, total) and gamma-glutamyl transferase levels

**Fig. 2:**
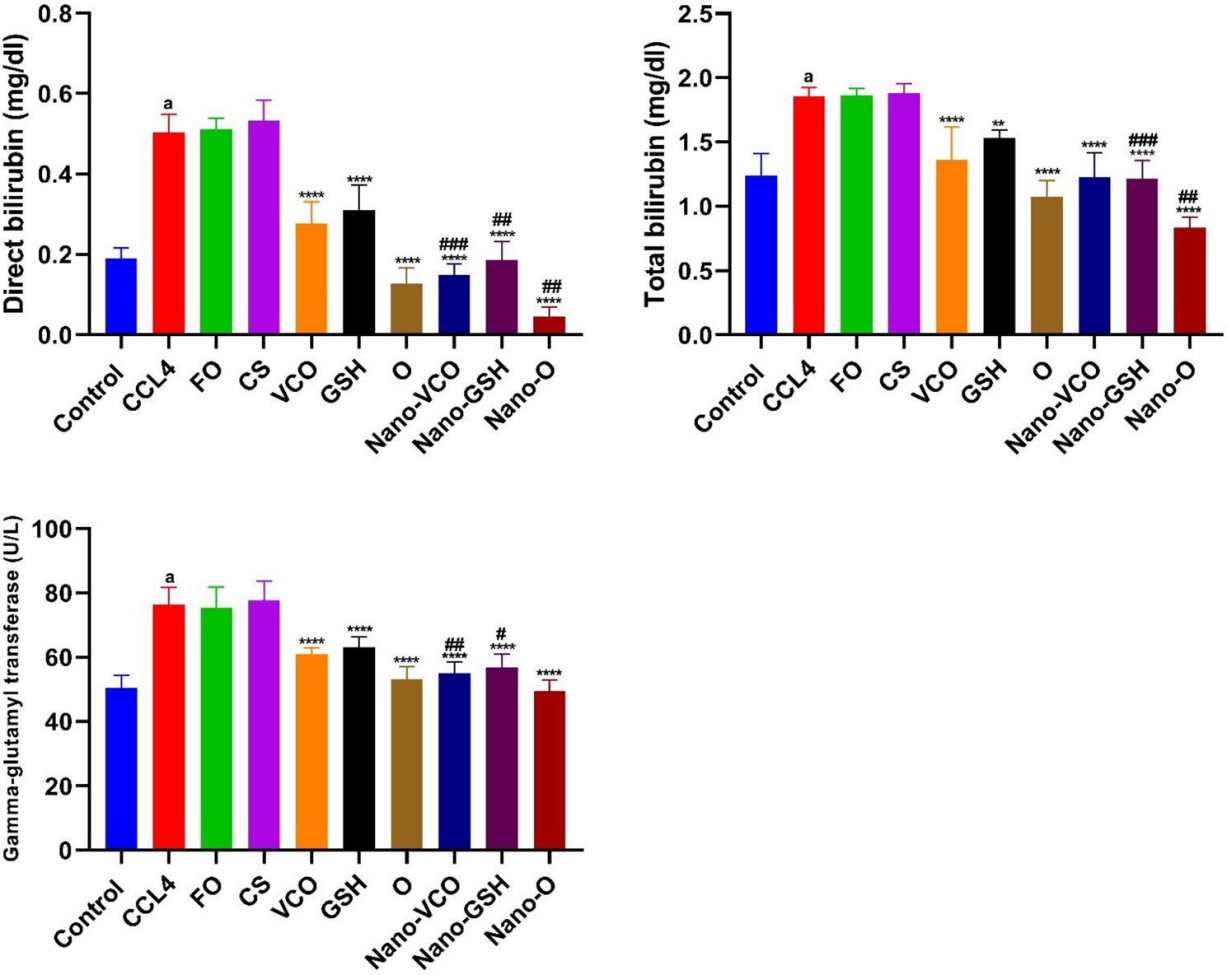
The impact of different forms of glutathione (GSH) and virgin coconut oil (VCO), including conventional, nanoparticle, and combination forms, on blood levels of bilirubin (direct and total) and gamma-glutamyl transferase in rats with acute liver failure induced by carbon tetrachloride (CCl_4_). Data are the means ± SEM (*n* = 6). ^a^*p* < 0.0001 as compared with the control group. **p < 0.01 and ****p < 0.0001 as compared to the CCl_4_-treated group. ^#^*p* < 0.05, ^##^*p* < 0.01 and ^###^*p* < 0.001 when contrasted with the related nanoparticle category. *Note FO* ferric oxide, *CS* chitosan, *Nano-VCO* virgin coconut oil nanoparticles, *Nano-GSH* glutathione nanoparticles, *O* virgin coconut oil-glutathione combinations, *Nano-O* virgin coconut oil-glutathione nanoparticle combinations

Compared to the negative control group, rats administered CCl_4_ revealed substantially higher levels of blood-direct bilirubin (df = 10, F = 3.234) (*p* < 0.0001), serum total bilirubin (df = 10, F = 5.513) (*p* < 0.0001), and GGT (df = 10, F = 1.830) (*p* < 0.0001). However, compared to the CCl_4_-induced liver failure group, rats treated with CS or Fe_3_O_4_ did not demonstrate noticeable increases in levels of blood-direct bilirubin (F_(2,15)_ = 0.7642) (*p* < 0.0001), total bilirubin (F_(2,15)_ = 0.2984) (*p* < 0.0001), or GGT (F_(2,15)_ = 0.2596) (*p* < 0.0001). Compared to the CCl_4_-induced liver failure group, both conventional and NP forms of VCO, GSH, or their combination resulted in a considerable decrease (*p* < 0.0001) in blood direct bilirubin (F_(6,35)_ = 70.19), total bilirubin (F_(6,35)_ = 25.46), and GGT (F_(6,35)_ = 32.59) levels. It is interesting that, compared to rats treated with their respective conventional medications, animals that were provided with NP versions of VCO or GSH, or their combination, demonstrated a considerable reduction in blood direct bilirubin, total bilirubin, and GGT, as indicated in (Fig. 2).

### The influence of coconut oil, glutathione, and their nanoparticle formulations on inflammatory cytokine levels (TNF-α and IL-1β) in rats with CCl4-induced liver failure

When compared with the negative control group, the rats who received CCl_4_ displayed a considerable elevation in the levels of tissue TNF-α (df = 10, F = 7.371), (*p* < 0.0001), and IL-1β (df = 10, F = 5.183), (*p* < 0.0001). However, when these results were contrasted with the CCl_4_-induced liver failure group, no noteworthy changes were found in the levels of tissue TNF-α (F_2,15_= 0.8731) and IL-1β (F_2,15_= 0.1304) in the rats treated with CS or Fe_3_O_4_.

**Fig. 3:**
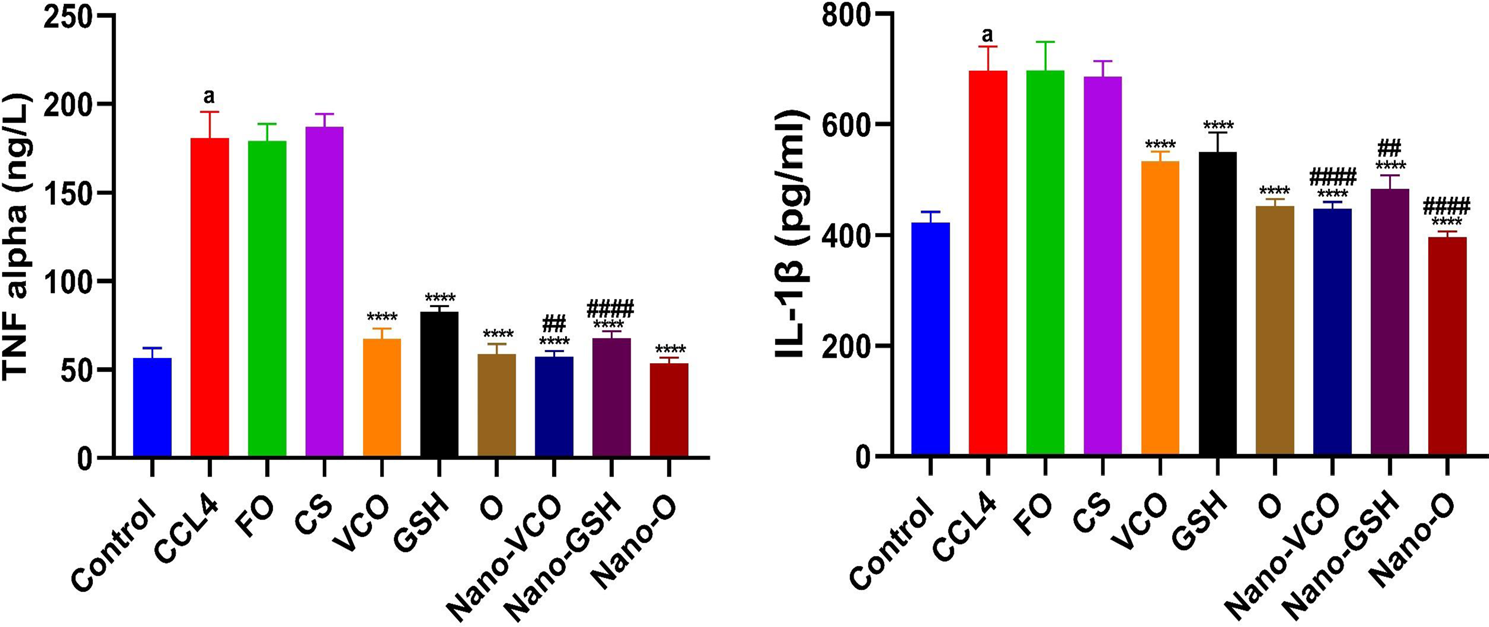
The impact of different forms of glutathione (GSH) and virgin coconut oil (VCO), including conventional, nanoparticle, and combination forms, on tissue levels of interleukin-1 beta (IL-1β) and tumor necrosis factor alpha (TNF-α) in rats with acute liver failure induced by carbon tetrachloride (CCl_4_). Data are the means ± SEM (*n* = 6). ^a^*p* < 0.0001 as compared with the control group. ****p < 0.0001 as compared to the CCl_4_-treated group. ^##^*p* < 0.01 and ^####^*p* < 0.0001 when contrasted with the related nanoparticle category. *Note FO* ferric oxide, *CS* chitosan, *Nano-VCO* virgin coconut oil nanoparticles, *Nano-GSH* glutathione nanoparticles, *O* virgin coconut oil-glutathione combinations, *Nano-O* virgin coconut oil-glutathione nanoparticle combinations

Contrary to the CCl_4_-induced liver failure group, a substantial decrease (*p* < 0.0001) in the levels of tissue TNF-α (F_(6,35)_ = 229.9) and IL-1β (F_(6,35)_ = 90.63) was found when either the conventional or NP forms of VCO, GSH, or their combination were provided to rats. NP versions of VCO or GSH, or their combination, significantly reduced tissue TNF-α and IL-1β levels in rats compared to those treated with conventional drugs (Fig. 3).

### The effect of coconut oil, glutathione, and their nanoparticle formulations on liver tissue malondialdehyde and GPx levels in rats with CCl4-induced liver failure

**Fig. 4:**
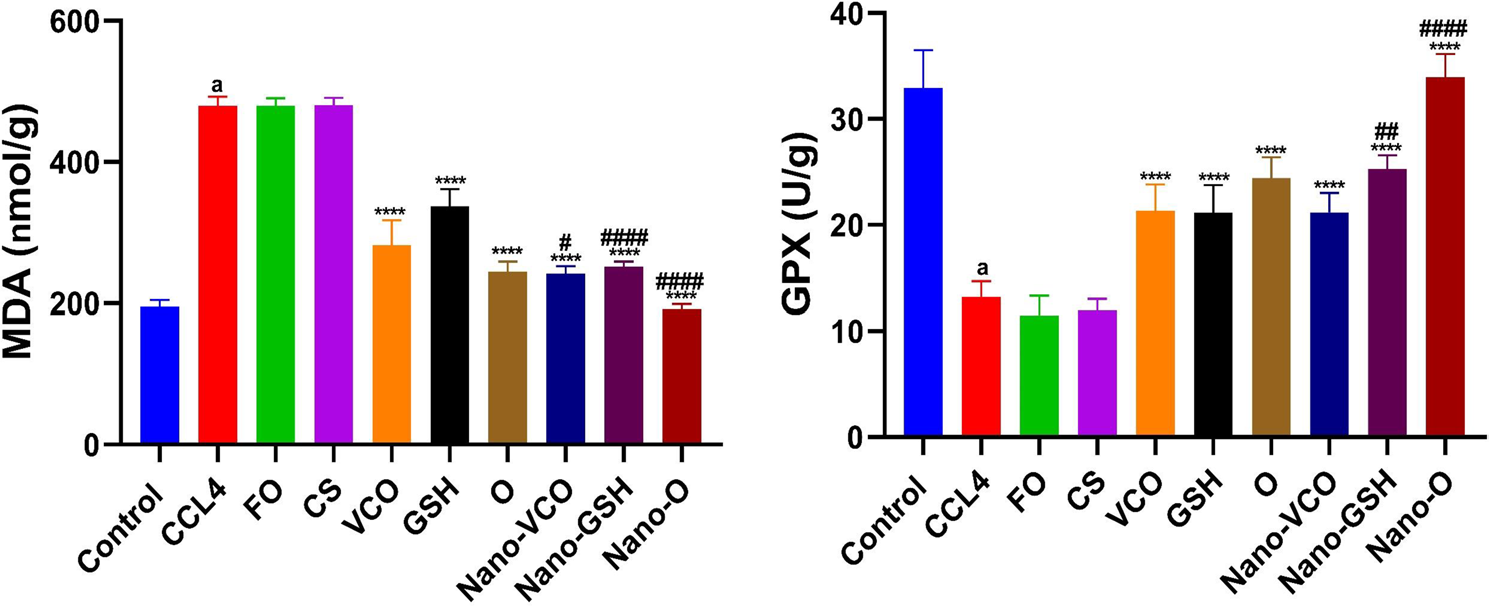
The impact of different forms of glutathione (GSH) and virgin coconut oil (VCO), including conventional, nanoparticle, and combination forms, on tissue levels of malondialdehyde (MDA) and glutathione peroxidase (GPx) in rats with acute liver failure induced by carbon tetrachloride (CCl_4_). Data are the means ± SEM (*n* = 6). ^a^*p* < 0.0001 as compared with the control group. ****p < 0.0001 as compared to the CCl_4_-treated group. ^#^*p* < 0.05, ^##^*p* < 0.01 and ^####^*p* < 0.0001 when contrasted with the related nanoparticle category. *Note FO* ferric oxide, *CS* chitosan, *Nano-VCO* virgin coconut oil nanoparticles, *Nano-GSH* glutathione nanoparticles, *O* virgin coconut oil-glutathione combinations, *Nano-O* virgin coconut oil-glutathione nanoparticle combinations

Rats treated with CCl_4_ revealed a considerable rise in tissue MDA levels (df = 10, F = 2.201), (*p* < 0.0001), and a significant drop in GPx levels (df = 10, F = 6.217), (*p* < 0.0001) compared to the negative control group, as indicated in (Fig. 4). Rats treated with CS and Fe_3_O_4_ did not demonstrate significant changes in MDA (F_(2,15)_ = 0.017) and GPx (F_(2,15)_ = 2.230) levels compared to the CCl_4_ group. When conventional therapies or NP forms of VCO, GSH, or their combination were administered, there was a significant drop in tissue MDA levels (F_(6,35_=156.9) (*p* < 0.0001). In contrast, GPx (F_(6,35)_ = 56.24) levels dramatically rose (*p* < 0.0001) compared to the CCl_4_-induced liver failure group. Compared to animals given the same conventional treatments, all groups treated with NPs showed a notable decrease in tissue MDA levels and a great rise in tissue GPx (Fig. 4).

### Histopathological evaluation

Analysis of H and E-stained slices from the control group revealed a histological image that closely resembled the typical structure of a healthy liver, with an intact central vein and hepatocytes arranged in regular, straight cords. Significant histopathological alterations are observed in the untreated group induced with CCl_4_, specifically in the liver tissue and blood vessels. The liver tissue cells display varying levels of damage. This might vary from the occurrence of ballooning (vacuolar) degeneration, characterized by inflated cells with clear cytoplasm, to necrosis, which refers to the death of cells. A notable characteristic is the occurrence of inflammatory cells invading the liver parenchyma. The presence of lymphocytes, macrophages, and plasma cells in these cells indicates an immunological response to liver injury.

**Fig. 5:**
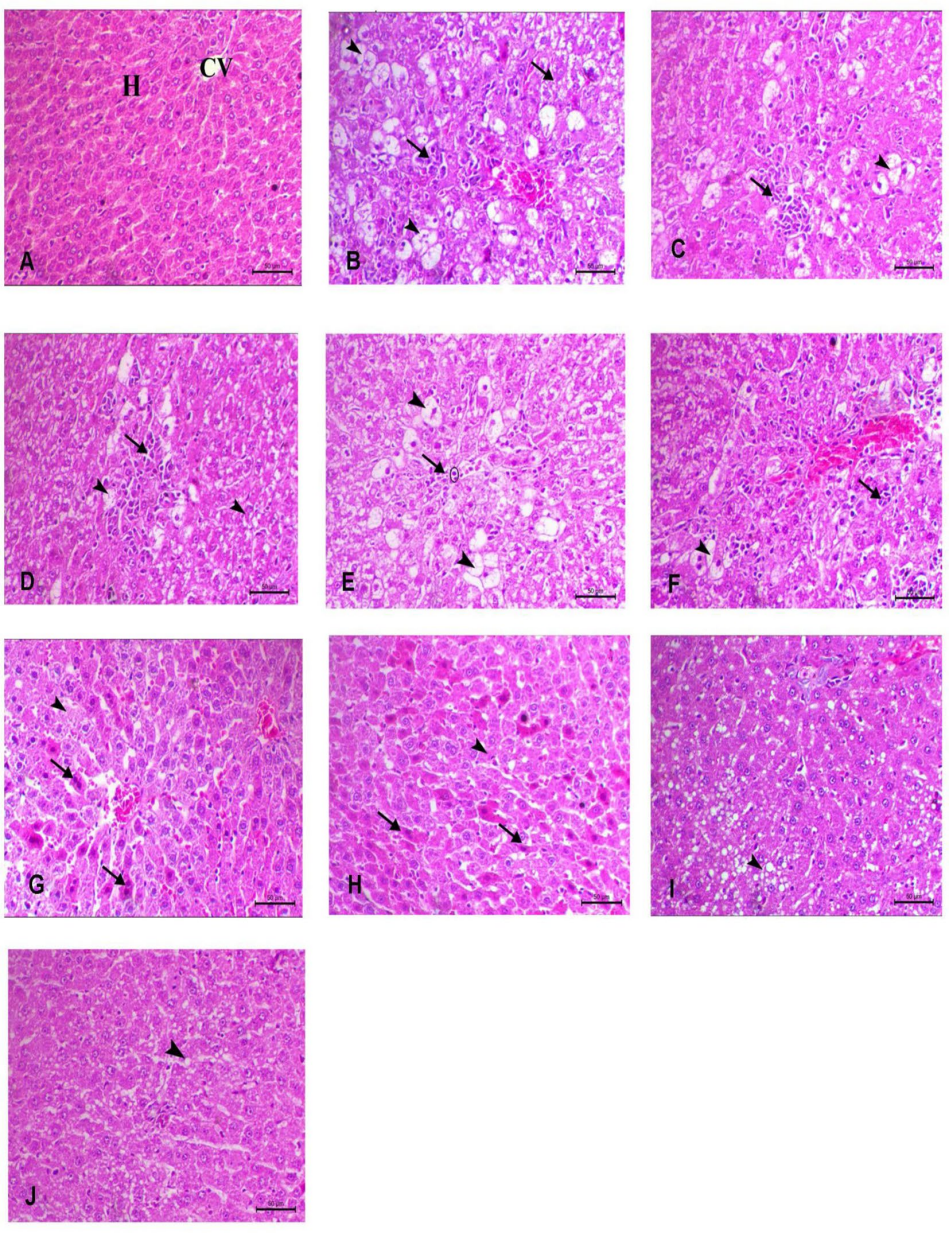
A representative image of liver tissue with HE staining **A** control group within normal structure of the liver lobules, central vein, and surrounding hepatocytes is clearly visible, **B** CCl_4_ group show moderate to severe vacuolar degeneration (arrowhead) and multifocal necrosis (yellow arrow) with multifocal inflammatory cell infiltration (black arrow) and hemorrhage in between hepatocyte (star), **C** and **D** CS and FO groups show focal hepatic necrosis mixed with mononuclear inflammatory cells (arrow) surrounded with hepatocytes reveal marked vacuolation (arrowhead), **E** and **F** represent GSH and VCO show mild improvement of liver tissue within focal hepatic necrosis mixed with mononuclear inflammatory cells and diffuse moderate vacuolar changes within the hepatocytes (arrowhead) and presence of mitotic figure (circle), **G**, **H** Nano-CS and Nano-VCO show more improvement degree of hepatic tissue with presence of mitotic figure and mild infammatory cells infilterated, **I** represents conventional combination (O) of treatments show normal hepatic parenchyma with midzonal area of fatthy changes in hepatic parenchyma. **J** that represent nano-combination (Nano-O) treatments show more healthy hepatic tissue within mild degree of periportal hepatic vacuolation consistent with mild fatty change (arrowhead). Scale bar = 50 μm. *CCl*_*4*_ carbon tetrachloride, *CS* chitosan, *GSH* glutathione, *Fe*_*3*_*O*_*4*_ ferric oxide, *VCO* virgin coconut oil

The normal arrangement of hepatocytes in cords is disrupted due to inflammation and cell damage. Neither the CS treatment nor Fe_3_O_4_ treatment showed any discernible protective effect against liver injury. Liver tissue health has mildly improved in the groups receiving GSH treatment, and VCO treatment. The normal arrangement of hepatocytes in cords is disrupted in some areas and normal in other areas, but the extent of inflammation appears somewhat limited in the form of some decrease of inflammatory cells. Observing areas with increased mitotic figures (dividing cells) and a decreasing level of vacuolar degeneration, which indicate decreased cell injury and regeneration of damaged cells. The Nano-GSH and Nano-VCO-treated group improved liver cells with the normal architecture and appeared to have more hepatocytes within mitotic figure and moderate congestion in blood vessels. The combination of treatments appeared to produce healthy hepatic tissue and healthy hepatocytes with a moderate level of fatty change. Following CCl_4_ exposure, treatment with nanoparticles restored normal liver architecture with very mild fatty changes (reversible degeneration) (Fig. 5) (Table 3).

**Table 3:** Summarizes each group’s liver tissue histopathology lesion scores

Lesion	Congestion of CV	Thrombosis	Hepatic necrosis	Hydrobic degeneration	Mononuclear cell infiltration
Group					
Control	-	-	-	-	-
CCL_4_	+++	++	+++	+++	+++
CS	++	++	+++	++	++
Fe_3_O_4_	++	++	+++	++	++
GSH	+	-	++	++	+
VCO	+	+	++	++	+
Nano-GSH	+	-	+	+	
Nano-VCO	+	-	+	+	
O	-	-	-	-	-
Nano-O	-	-	-	-	-

- No lesions, + lesions present in 2–3 sections, ++ lesions present in 4–7 sections and +++ lesions present in 8–10 sections

*CCL*_*4*_ carbon tetrachloride, *CS* chitosan, *GSH* glutathione, *Fe*_*3*_*O*_*4*_ ferric oxide, *O* combination of GSH and VCO, *VCO* virgin coconut oil

### Immunohistochemical analysis

**Fig. 6:**
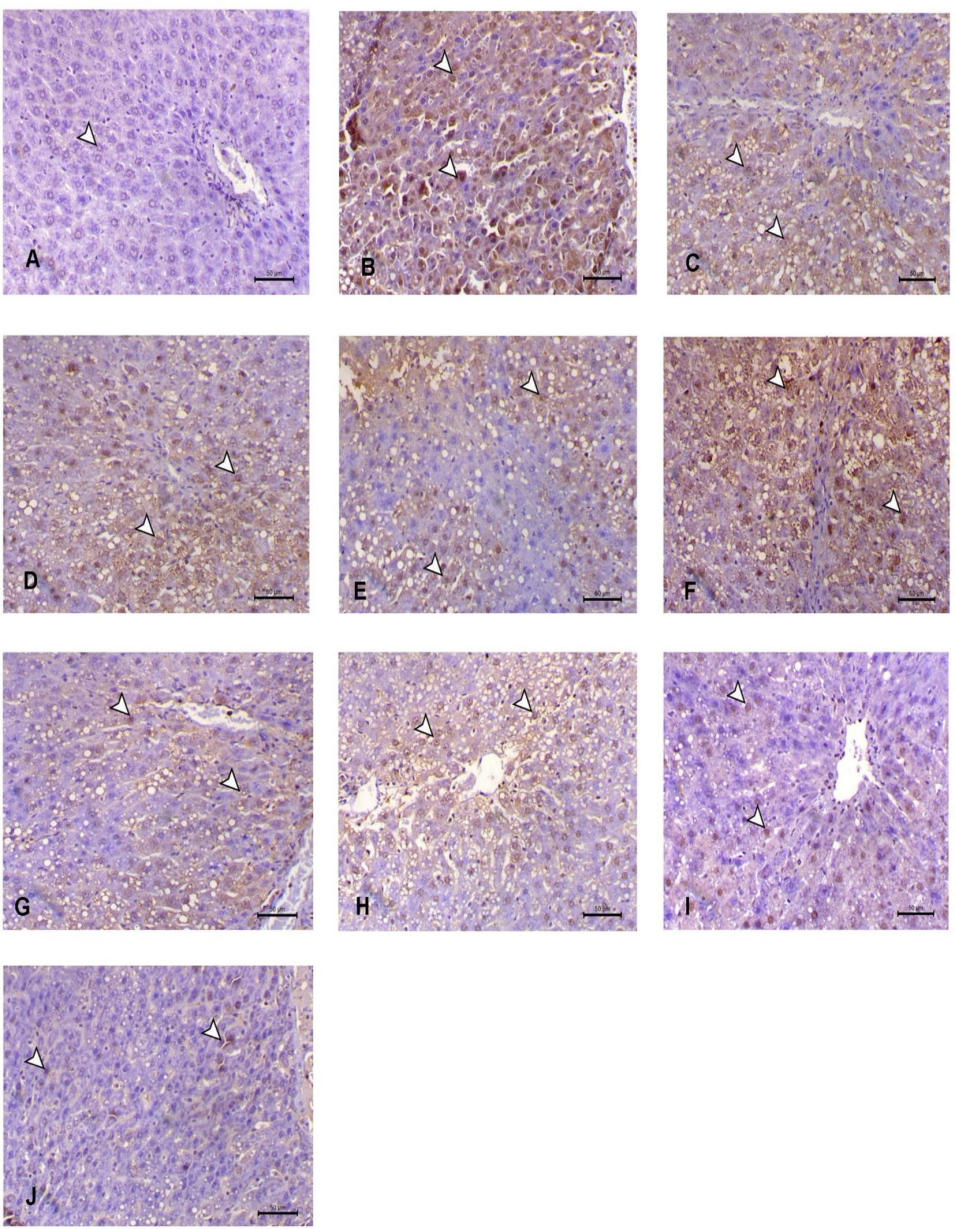
Immunohistochemical analysis revealed **A** the absence of NF-κB expression in the liver tissue of the control group. **B** CCl_4_ treatment significantly increased NF-κB expression in the cytoplasm and nucleus of hepatocytes, as indicated by brown staining (arrowhead). **C** and **D** CS and FO groups showing less increase in NF-κB immunostaining. **E** and **F** showing decrease of NFκB-P65 antibody within the hepatic cells in treated groups with GSH, and VCO. **G**, **H** treatment groups with Nano-GSH and Nano-VCO showed an obvious decrease in both cytoplasmic and nuclear expression of the NFκB-P65 antibody (arrowhead). **I** The combined treated group (O) showed a marked decrease in cytoplasmic and nuclear expression of the NFκB-P65 antibody (arrowhead). **J** The nano combined-treated group (Nano-O) showed a significant decrease in NFκB-P65 immunostaining within the hepatic cells (arrowheads). *CCL*_*4*_ carbon tetrachloride, *CS* chitosan, *GSH* glutathione, *Fe*_*3*_*O*_*4*_ ferric oxide, *VCO* virgin coconut oil

The examination of rat livers using immunohistochemical analysis demonstrated a notable augmentation in NF-κB expression subsequent to CCl_4_ therapy. When GSH and VCO were given together with CCl_4_, there was a partial decrease in NF-κB expression. The treatment of Nano-GSH or Nano-VCO produced a more marked decrease of NF-κB expression compared to single treatment. Interestingly, the combined traditional treatment led to a significant decrease in both nuclear and cytoplasmic expression of NF-κB antibody, which differed from the NPs combination treatment groups where expression was completely prevented, similar to the control group (Fig. 6) (Table 4).

**Table 4:** Effects of conventional glutathione, virgin coconut oil and their nano form alone and in combination on the percentage expression of nuclear factor-κB (NF-κB), in the liver of rats exposed to CCL_4_ hepatotoxicity

Groups	Control	CCL_4_	Fe_3_O_4_	CS	GSH	VCO	O	Nano-GSH	Nano-VCO	Nano-O
**NF-κB**	4.607±0.3811	72.04±1.696^a^	67.06±0.9757^N^	65.64±1.620^N^	36.01±1.668****	43.23±1.330****	16.89±0.7239****	24.90±0.7717****^###^	29.26±1.897****^####^	9.813±0.8599****^#^

*CCL*_*4*_ carbon tetrachloride, *Fe*_*3*_*O*_*4*_ ferric oxide, *CS* chitosan, *GSH* glutathione, *VCO* virgin coconut oil, *O* combination of GSH and VCO

^a^Value means there is significant difference between control group and CCL_4_ group at *P* < 0.0001

^N^Value means there is no significant difference between CCL_4_ group and Fe_3_O_4_ group or CS group

****Value means there is significant difference between CCL_4_ group and treated groups at *P* < 0.0001

^###, ####^ values mean there is significant difference between the conventional treated group and its nano form treated groups at *P* < 0.001 and *P* < 0.001, respectively

## Discussion

Liver failure can result from virus infection, excessive drug usage, alcohol addiction, and exposure to other toxic substances [43]. The hepatotoxic experimental rat model of CCl_4_ destruction replicates physiological and pathological human liver injury [44]. Compared to the negative control group, CCl_4_ administration significantly raised blood AST, ALT, ALB, ALP, serum direct bilirubin, total bilirubin, and GGT. This is consistent with CCl_4_’s documented hepatotoxicity. Weber, Boll [45] revealed that CCl_4_ destroys hepatocytes, releasing these enzymes into the blood. Research by Kovalovich, DeAngelis [46] and Domitrović, Jakovac [47] reveals that CCl_4_-induced liver injury leads to raised blood bilirubin levels due to poor conjugation and excretion and increased GGT activity, resulting in oxidative stress, respectively. While CCL_4_ promotes hypoproteinemia, our study found hyperalbuminemia, potentially attributable to the fact that the liver is not the exclusive site of albumin synthesis; it can also be produced in extrahepatic tissues such as the intestine, kidney, pancreas, brain, and reproductive organs [48, 49].

Increased tissue TNF-α, IL-1β, MDA, and reduced GPx levels were detected in CCl_4_-treated rats compared to the control group. A previous study supports this. Xiao, Liong [50] observed that CCl_4_ administration in rats raises hepatic TNF-α and IL-1β levels, contributing to acute liver injury inflammation. Moreover, Recknagel, Glende Jr [51] and Szymonik-Lesiuk, Czechowska [52] revealed that CCl_4_ metabolism generates free radicals, which cause liver tissue lipid peroxidation, higher MDA levels, and decreased antioxidant enzyme activity, including GPx, respectively.

The study also showed that the tissues of rats that were given CCl_4_ had serious problems, such as ballooning degeneration, necrosis, and inflammatory cell infiltration. CCl_4_ administration resulted in a significant increase in NF-κB expression, as demonstrated by immunohistochemical investigation, these observations are consistent with past research. Boll, Lutz [53] found similar histological abnormalities in CCl_4_-induced liver injury, including centrilobular necrosis and inflammatory cell infiltration. Luedde and Schwabe [54] found that CCl_4_-induced liver injury enhances NF-κB activation, contributing to the inflammatory response and cell death.

The present investigation demonstrated the considerable hepatoprotective effects of GSH, VCO, and their NP formulations in a rat model of CCl_4_-induced ALF. The investigation indicated that administration of these medications, either alone or in combination, efficiently restored the elevated levels of blood AST, ALT, ALB, ALP, GGT, and bilirubin (direct and total) to normal, suggesting their capacity to attenuate CCl_4_-induced liver impairment. The hepatoprotective effects of GSH and VCO can be attributed to their potent antioxidant and anti-inflammatory actions. This is consistent with past studies revealing the antioxidant properties of GSH [55–57]. VCO, on the other hand, is rich in medium-chain fatty acids and polyphenolic compounds that display high antioxidant and anti-inflammatory properties [58–60].

Interestingly, the NP formulations of GSH and VCO demonstrated stronger hepatoprotective effects compared to their conventional counterparts. This can be attributable to the enhanced bioavailability, targeted dosing, and sustained release properties of the nanoparticles [61–63]. The nanoparticle carriers can improve the solubility, stability, and tissue-specific accumulation of GSH and VCO, leading to more effective attenuation of CCl_4_-induced liver injury. The synergistic benefit demonstrated by the combination of VCO and GSH NPs suggests a potential for multi-targeted therapy in ALF management.

These findings correlate with earlier research on the hepatoprotective effects of VCO and GSH. For instance, a study by Zakaria, Rofiee [64] proved the capacity of VCO to lower hepatic enzyme levels in rats with paracetamol-induced liver injury. Similarly, Honda, Kessoku [65] showed the efficacy of GSH in improving liver function markers in people with non-alcoholic fatty liver disease.

The substantial reduction in tissue levels of TNF-α and IL-1β with NP formulations of VCO and GSH, especially in combination, indicates their powerful anti-inflammatory activities. Inflammation plays a crucial role in ALF pathophysiology, and these pro-inflammatory cytokines are critical mediators of liver injury [66].

The enhanced efficacy of NP formulations in reducing these cytokines compared to conventional forms suggests improved cellular absorption and targeted distribution to inflamed regions. This is consistent with past research indicating the anti-inflammatory potential of both VCO and GSH [67, 68]. These findings are particularly remarkable given the prominent role of inflammation in propagating liver injury and leading to systemic effects in ALF. By successfully suppressing these pro-inflammatory cytokines, the NP formulations may help to halt the cycle of inflammation and hepatocellular damage, thereby improving outcomes in ALF [69].

Nanoparticle formulations of VCO and GSH exhibit potent antioxidant effects, as evidenced by a significant decrease in tissue MDA levels and a rise in GPx activity. Oxidative stress is a critical aspect in the pathogenesis of CCl_4_-induced liver injury and ALF in general [70]. This is consistent with past research indicating the antioxidant properties of both VCO and GSH [71, 72]. The synergistic benefit reported with the combo therapy implies a holistic approach to managing oxidative stress in ALF. By efficiently lowering lipid peroxidation (as indicated by reduced MDA levels) and improving antioxidant defense mechanisms (as shown by enhanced GPx activity), the NP formulations may help attenuate oxidative damage and promote liver regeneration [73].

Histological investigation provides crucial visual verification of the NP formulations’ hepatoprotective advantages. The transition from severe liver damage in the CCl_4_-induced ALF group to near-normal hepatic architecture in the NP combo therapy group indicates the outstanding efficacy of this therapeutic strategy.

The observed reduction in inflammatory cell infiltration, lower vacuolar degeneration, and higher mitotic figures in hepatocytes with NP treatments, particularly in combination, corroborate the biochemical findings. These histological improvements demonstrate that the NP formulations not only guard against new damage but also promote liver regeneration. These observations are consistent with recent research on the hepatoprotective effects of VCO and GSH [17, 74].

The immunohistochemical investigation of NF-κB expression gives critical insights into the molecular mechanisms underpinning the hepatoprotective advantages of the NP formulations. The substantial reduction in NF-κB expression with NP treatments, notably the entire protection found with the combo therapy, indicated potent anti-inflammatory and antioxidant effects at the molecular level.

NF-κB is a key transcription factor involved in the modulation of inflammatory responses and oxidative stress [75]. These findings are consistent with past research indicating the capacity of both VCO and GSH to impact NF-κB signaling [76, 77]. The enhanced efficacy of NP formulations in reducing NF-κB activation compared to conventional forms further highlights the advantages of NP-based medication delivery in targeting molecular pathways involved in liver injury.

In conclusion, this work provides significant evidence for the efficacy of NP formulations of virgin coconut oil and glutathione in treating acute liver failure in a rat model. The higher efficacy of these NP formulations compared to conventional forms across numerous criteria, including liver function markers, inflammatory cytokines, oxidative stress indicators, and histological features, highlights their promise as a novel therapeutic method for ALF. Future research should focus on identifying the precise mechanisms of action of these NP formulations, improving their composition and administration, testing their efficacy in larger animal models, and finally, conducting clinical trials. Additionally, studying the long-term safety and potential negative effects of these NP formulations will be vital for their application in clinical practice.

This discovery offers up new options for the development of nanoparticle-based therapeutics for liver diseases, possibly altering the therapy landscape for acute liver failure and other hepatic conditions.

## Limitation section

This study offers significant insights into the therapeutic efficacy of NP formulations of GSH and VCO in a rat model of CCl_4_-induced ALF, although many limitations must be recognized: The research employed a rat model to generate ALF, which, although widely recognized, may not entirely mimic the intricacies of human liver failure. Extended investigations are essential to evaluate the sustainability of treatment effects and the possibility of long-term liver regeneration and recovery. An increased sample size of rats, incorporating both sexes, could yield more reliable data.

## Electronic supplementary material

Below is the link to the electronic supplementary material.

Supplementary Material 1

Supplementary Material 2

## Data Availability

No datasets were generated or analysed during the current study.

## Abbreviations

ALF: Acute liver failure
ALT: Alanine aminotransferase
ALB: Albumin
ALP: Alkaline phosphatase
AST: Aspartate Aminotransferase
CCl_4_: Carbon tetrachloride
CS: Chitosan
FTIR: Fourier transform infrared spectroscopy
GGT: Gamma-glutamyl transferase
GSH: Glutathione
GPx: Glutathione peroxidase
IL-1β: Interleukin 1 beta
MDA: Malondialdehyde
NP: Nanoparticle
NF-κB: Nuclear factor-kappa B
TEM: Transmission electron microscopy
TNF-α: Tumor necrosis factor alpha
VCO: Virgin coconut oil
XRD: X-ray Diffraction Spectroscopy
