# Supplementary figures and images for: Evaluation of the therapeutic potential of novel nanoparticle formulations of glutathione and virgin coconut oil in an experimental model of carbon tetrachloride-induced liver failure
Source: BMC Pharmacol Toxicol. 2024 Oct 8;25:74. doi: 10.1186/s40360-024-00795-x (PMC11460069; doi:10.1186/s40360-024-00795-x)

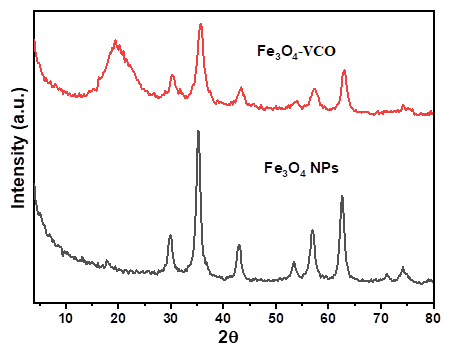


**Figure S1.**


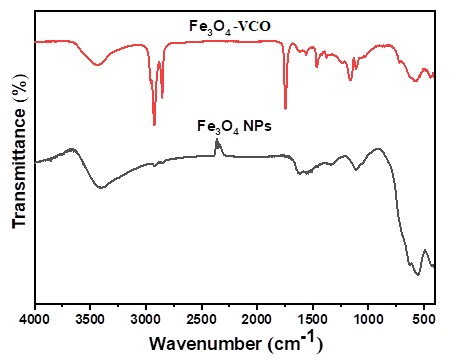


**Figure S2.**


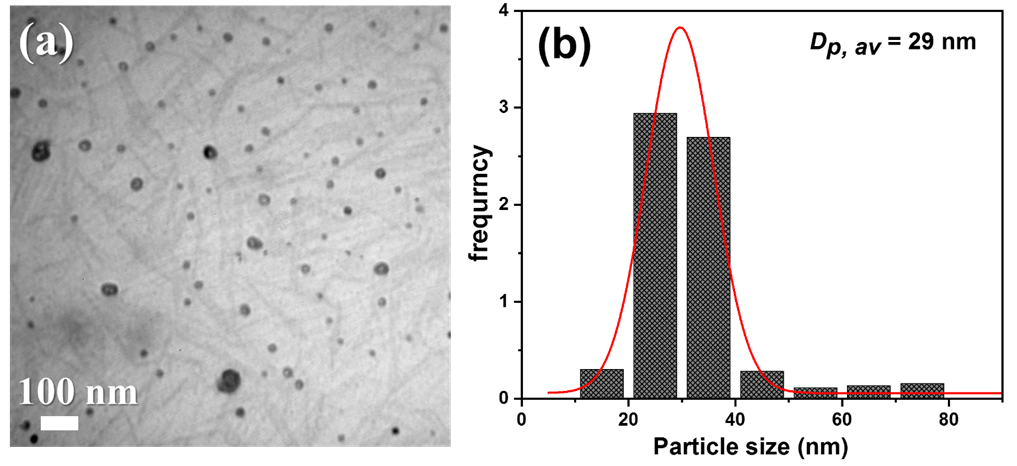


**Figure S3**


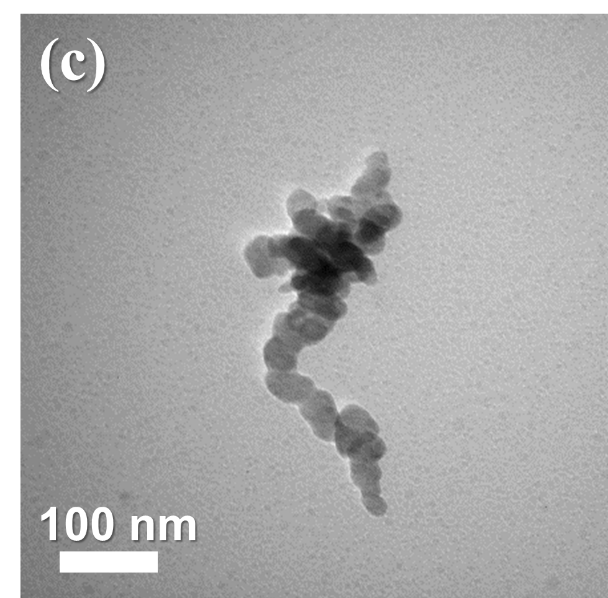


**Figure S4**

Supplement: Supplementary file 2 — Supplementary Material 2 [file 40360_2024_795_MOESM2_ESM.docx]
